# Large Language Model–Assisted Radiology Report Quality Assurance: Real-World Multicenter Evaluation

**DOI:** 10.2196/106250

**Published:** 2026-10-28

**Authors:** Lujing Wang, Yushuang Zhang, Liang Peng, Pei Liu, Li Wang, Yu Cui, Yixin Chen, Xingyu Li, Yitian Tao, Xiuxian Gong, Xiaoying Zhao, Yujiao Song, Jian Chen, Shihua Zhao, Fuying Wang, Jian Ma, Xinxiang Zhao

**Affiliations:** 1 Department of Radiology, The Second Affiliated Hospital of Kunming Medical University, Kunming, Yunnan, 650101, China, 86 0871-63402918; 2 Department of Radiology, The Sixth Affiliated Hospital of Kunming Medical University, Yuxi, Yunnan, China; 3 School of Information and Software Engineering, University of Electronic Science and Technology of China, Chengdu, Sichuan, China; 4 School of Biomedical Engineering, ShanghaiTech University, Shanghai, China; 5 Department of Radiology, School of Clinical Medicine, University of Cambridge, Cambridge, United Kingdom; 6 Department of Radiology, National Center for Cardiovascular Diseases, Fuwai Hospital, Beijing, China; 7 Department of Radiology, Stanford Center for Artificial Intelligence in Medicine and Imaging, Stanford University, Stanford, CA, United States

**Keywords:** large language model, radiology report, quality assurance, clinical AI, report error, external validation, human evaluation, artificial intelligence, AI

## Abstract

**Background:**

Radiology reports serve as the definitive clinical record of imaging diagnoses, with their accuracy being directly critical to patient safety. However, amid escalating workloads, manual review remains inadequate for capturing subtle errors in reports on a large scale. Although large language models (LLMs) have demonstrated considerable promise in medical text processing, previous studies have predominantly been confined to radiology report text from a single imaging modality or datasets with synthetic errors.

**Objective:**

This study aimed to evaluate the feasibility, accuracy, and generalizability of general-purpose LLMs for quality assurance of radiology report text from multiple imaging modalities (x-ray, computed tomography [CT], and magnetic resonance imaging [MRI]), multi-anatomical regions, and multicenter clinical settings, with head-to-head comparisons conducted against radiologists of varying seniority.

**Methods:**

Radiology report texts from 3 aforementioned imaging modalities and 5 anatomical regions (head, chest, abdomen, spine, and knee) were retrospectively collected from center 1 (March 2025-May 2025) and reviewed using a clinician-annotated 5-category error taxonomy (type 1: interpretive; type 2: factual). Five LLMs (Qwen3, GPT-5.4 Pro, Gemini 3.1 Pro, Llama 4, and DeepSeek-V3.1) were benchmarked on 10,000 randomly selected reports, and the best-performing model was evaluated on the full cohort. A blinded head-to-head comparison with 3 radiologists (3-10 years of experience) used a modality-stratified, error-enriched challenge set. The radiologists subsequently rated the model-generated explanations using a 5-point Likert scale. Cross-center performance was evaluated in an independent external cohort. The same 2 senior radiologists labeled reports from both centers using the same protocol, resolving disagreements by consensus.

**Results:**

In total, 4.6% (4431/95,582) of the reports in center 1 contained at least one confirmed text internal error. Qwen3, the selected model, achieved a report-level sensitivity, specificity, positive predictive value, and *F*_1_-score of 0.99, 0.99, 0.90, and 0.94, respectively, with high performance across reports from the 3 imaging modalities and multiple anatomical regions and better detection of factual than interpretive errors. In the error-enriched challenge set, its error instance exact category recall exceeded that of the radiologists (0.96 vs 0.45-0.67; *P*<.001), with a shorter mean task completion time (5.80 vs 55.80-67.80 seconds per report). Explanations had a mean Likert score of 4.57, and all inter- and intraobserver intraclass correlation coefficients exceeded 0.89. In center 2, balanced accuracy was 0.94 (95% CI 0.92‐0.95). Before consensus, Cohen κ for report-level error presence was 0.783 (center 1) and 0.850 (center 2).

**Conclusions:**

This study suggests that LLMs can serve as efficient adjunctive tools for text-level quality control of radiology reports, particularly for detecting explicit factual errors. Performance remained stable across centers. In the text-only review task, the model achieved higher exact category recall and shorter task completion times than the participating radiologists. These findings support the potential value of LLM-assisted radiology report text review in real-world clinical environments.

## Introduction

Radiology reports serve as critical determinants of clinical decision-making, with their accuracy directly influencing patient outcomes and health care safety. However, the integrity of these reports is persistently threatened by human errors [1]. In recent years, the workload of radiologists has increased significantly [2,3]. Radiology departments in Chinese tertiary hospitals generate over 10,000 imaging reports daily, and senior radiologists review an average of over 100 reports each day. In this high-pressure clinical environment, factors such as work stress, varying professional expertise, and fatigue drive error rates as high as 15% to 20%. Of these errors, 3% to 5% pose tangible risks of diagnostic delays or iatrogenic harm [3,4]. Despite established quality assurance protocols, manual double reading remains resource intensive, time-consuming, and imperfect: even experienced radiologists miss up to 10% of subtle yet clinically consequential errors [5]. Thus, developing efficient and accurate methods for detecting errors in radiology reports has become a critical challenge for improving imaging diagnostic quality.

Recent advancements in large language models (LLMs) have introduced a paradigm shift in medical text processing [6,7]. Unlike conventional deep learning models constrained by narrow applicability, LLMs demonstrate remarkable contextual comprehension, multimodal semantic alignment, and few-shot learning capabilities [8,9]. These strengths position LLMs to address complex challenges in health care text analysis [10,11], including the extraction of key information [12] and error detection in radiology reports [13-15]. Preliminary studies have validated LLMs’ proficiency in medical terminology processing and report summarization [16-18]. However, their application in radiology remains nascent, with existing research confined to single-modality reports (eg, chest x-rays) and synthetic error datasets [9,19-21]. No study has yet systematically evaluated LLMs’ performance across multimodal (x-ray, computed tomography [CT], and magnetic resonance imaging [MRI]) and multisite clinical settings using expert-annotated real-world error corpora.

This study focused on radiology reports from 3 imaging modalities (x-ray, CT, and MRI) covering multiple anatomical regions. We constructed a large-scale dataset of real-world radiology reports with expert-annotated, clinically relevant error types. We evaluated the potential of LLMs for automated error detection in authentic clinical reports. We conducted a comprehensive AI-human benchmarking study via blinded comparisons with radiologists of varying experience levels—senior, intermediate, and junior. We further assessed the robustness and generalizability of the top-performing LLM on an independent, external-center dataset of real erroneous reports. Our work aims to provide an intelligent solution for radiology report quality control; offer methodological insights for deploying LLMs in specialized clinical settings; and, ultimately, support improvements in diagnostic quality and patient safety.

## Methods

### Study Population and Dataset

The study design is summarized in Figure 1. Radiology report texts from 3 imaging modalities (x-ray, CT, and MRI) and 5 anatomical regions (head, chest, abdomen, spine, and knee) were retrospectively collected from center 1 (the Second Affiliated Hospital of Kunming Medical University) between March 2025 and May 2025. Inclusion criteria were (1) complete reports containing imaging modality, anatomical location, descriptive findings, and diagnostic conclusion; and (2) exclusion of emergency or functional imaging studies. All reports were initially drafted by junior radiologists (residents to attending physicians with 1‐7 years of experience) and subsequently reviewed by senior radiologists (associate chief physicians or above with ≥8 years of experience) as part of routine clinical workflow. All radiology reports were exported from the picture archiving and communication system and deidentified. We removed patient names, sexes, ages, inpatient numbers, accession numbers, contact details, addresses, dates of birth, examination dates, institution names, physician names, and any other direct or indirect identifiers. The retained data comprised the imaging modality, examination item, imaging findings, and diagnostic impression. Each record was assigned a randomly generated study ID in place of its original identifier. A linkage file connecting the study IDs to the original patient records was retained only within a restricted institutional environment. This file was not provided to any model or third-party platform.

**Figure 1.**
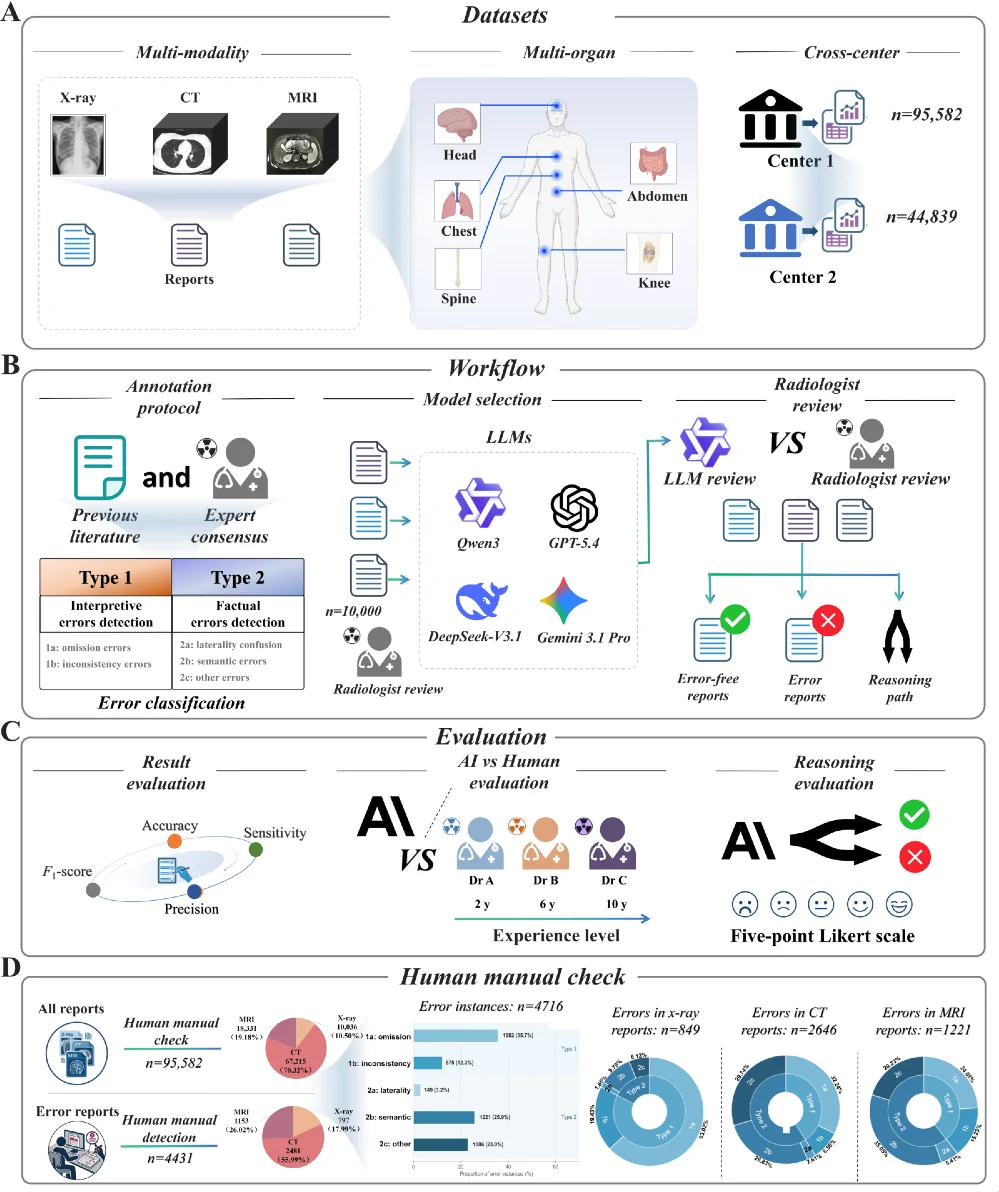
Study workflow. (A) Dataset: real radiology reports from 3 imaging modalities, multiple anatomical sites, and multiple centers. (B) We built a standardized error system. First, 10,000 reports tested large language models (LLMs)—Qwen3 (the best performing) was selected. (C) We evaluated Qwen3’s error detection performance in radiology reports from different dimensions, compared it with that of 3 radiologists (varied experience), and analyzed its reasoning via a 5-point Likert scale. (D) Distribution of reports and true error subtypes in the final cohort. CT: computed tomography; MRI: magnetic resonance imaging.

### Error Taxonomy and Annotation Protocol

Error annotations adhered to a standardized taxonomy established through previous literature and expert consensus [1,9] (Table 1). Radiology report errors were classified into five mutually exclusive categories: (1) omission (a finding documented in the imaging findings that was absent from the diagnostic impression or a diagnosis in the diagnostic impression that lacked any corresponding description in the imaging findings); (2) inconsistency (logical contradiction between description and impression [eg, “clear fracture line” is described, but the diagnosis states “no evidence of fracture”]); (3) laterality confusion (incorrect anatomical side or orientation [eg, “right” mislabeled as “left”]); (4) nonlogical errors due to inadvertent insertion of incorrect terms, including word misuse, typographical errors, irrelevant content, or lexical substitution; and (5) other errors (technical or formatting mistakes not covered above, such as impression numbering errors, unit mislabeling [eg, centimeters vs millimeters], or punctuation-induced ambiguity). Reports without errors were labeled as “N.” Errors 1 and 2 were grouped as type 1 (interpretive errors), whereas errors 3 to 5 constituted type 2 (factual errors). All 5 error categories were defined as text internal errors identifiable from the radiology report text alone. The taxonomy did not include imaging findings that were absent from the entire report and could be identified only through direct review of the source images. All reports were independently reviewed by 2 senior radiologists with 18 and 21 years of clinical experience to establish the report text reference standard labels. Discrepancies were resolved through consensus adjudication. The reviewers assessed only the report text and had no access to the corresponding source images, patient clinical records, or LLM outputs.

**Table 1.** Classification and definitions of errors in radiology reports.

Nature and error type	Definition	Example
Type 1: interpretive errors		
Omission	A finding documented in the imaging findings that is absent from the diagnostic impression or a diagnosis in the diagnostic impression that lacks any corresponding description in the imaging findings	Unreported known lesions (eg, the “right upper lobe posterior segment nodule” in the diagnostic impression is not mentioned in the imaging findings)
Inconsistency	Logical contradiction between description and impression, undermining report credibility	The description states “clear fracture line,” whereas the impression concludes “no definite fracture”
Type 2: factual errors		
Laterality confusion	Incorrect anatomical side or orientation, risking misdirected intervention	“Right tibial fracture” documented as “left”; “lateral meniscus tear” misreported as “medial”
Semantic error	Nonlogical errors due to inadvertent insertion of incorrect terms, including word misuse, typographical errors, irrelevant content, or lexical substitution	Redundant content insertion; misspelling (eg, “calcification spot” as “calcification zhuang”)
Other error	Technical or formatting mistakes not covered above	Misnumbered impression (eg, “Finding 1” labeled as “Finding 3”); unit error (eg, “5 mm” recorded as “5 cm”); ambiguous punctuation

### LLM Selection and Configuration

Five LLMs were evaluated: Qwen3-2507 (Alibaba Cloud; hereafter referred to as “Qwen3”), GPT-5.4 Pro (OpenAI), Gemini 3.1 Pro (Google DeepMind), Llama 4 (Meta AI), and DeepSeek-V3.1. All models were accessed via an OpenAI-compatible cloud API end point provided by GeekAI [22] and initially benchmarked using a randomly selected subset of 10,000 expert-annotated reports comprising 3000 x-ray reports, 4000 CT reports, and 3000 MRI reports. The LLM that demonstrated optimal performance was selected as the primary model for comprehensive analysis. The temperature parameter, which controls output randomness, was set to 0.1 to prioritize deterministic, conservative outputs aligned with the clinical imperative of minimizing false negatives. The models received only the textual content of the radiology reports; no medical images or other input modalities were provided. “X-ray,” “CT,” and “MRI” indicate the imaging examinations from which the report texts originated. The radiology report quality check prompt and translated report error detection examples are shown in Multimedia Appendix 1. Radiology report quality control was performed using Qwen3.

### Human and AI Comparative Assessment

A blinded, head-to-head evaluation was conducted to compare error detection and categorization performance between radiologists and the AI model. This study used a challenge set comprising 300 randomly selected reports (100 each from x-ray, CT, and MRI examinations) drawn from the expert-confirmed error-positive reports in the center 1 cohort. Three radiologists with differing experience levels participated, including Drs A (10 years), B (6 years), and C (3 years). These radiologists independently reviewed the reports for errors without access to source images, clinical history, reference labels, or model outputs. Participants were instructed to identify all errors and assign each to one of 5 prespecified taxonomy categories. Prior to the formal evaluation, all radiologists were trained on the taxonomy definitions and examples (Table 1) and completed a pilot session with 5 reports, which were excluded from the final analysis. Error detection assessments were locked upon submission. No further modifications were allowed. Review time was recorded, which was defined as the time required to complete the experimental text-only report auditing task for each report modality.

Report-level error detection sensitivity was defined as the proportion of error-containing reports in which at least one error was identified. Report-level exact set matches were counted as correct only when the complete set of predicted error categories exactly matched the adjudicated reference set; missed categories and additional incorrect categories were counted as failures. Error instance–level exact category recall was calculated as the number of ground truth error instances assigned the correct category divided by the total number of ground truth error instances.

### Interpretability and Clinical Reasoning Evaluation

To evaluate the clinical interpretability and reasoning validity of error detections generated by Qwen3, a total of 3 radiologists (Drs A, B, and C) independently rated the model’s explanatory rationales for errors identified in 300 radiology reports (100 each from x-ray, CT, and MRI examinations) using a 5-point Likert scale [23] (5=“strongly agree,” 4=“agree,” 3=“neutral,” 2=“disagree,” and 1=“strongly disagree”). The explanation rating task was performed separately after error detection. The explanations were presented without model identifiers, and the radiologists were blinded to their origin. The final score for each report was defined as the mean of the 3 reviewers’ ratings. Approximately 4 months after the initial assessment, the same radiologists re-evaluated a subset of reports. The subset consisted of 30 randomly selected reports for each of the 3 imaging modalities. The original scoring criteria were used. Reports were presented in a new random order. Radiologists were blinded to the scores of other reviewers.

### Cross-Center Data

To evaluate the robustness of Qwen3 and its cross-institutional generalizability, we conducted external validation on an independent dataset of 44,839 real-world radiology reports retrospectively collected from center 2 (the Sixth Affiliated Hospital of Kunming Medical University). The dataset included reports from the same 3 imaging modalities (x-ray, CT, and MRI) and covered major anatomical sites, including head, chest, abdomen, and spine. The same 2 senior radiologists independently labeled reports from both centers using the same prespecified taxonomy and annotation protocol.

### Statistical Analysis

Primary performance metrics included accuracy, balanced accuracy, sensitivity, specificity, precision, positive predictive value, negative predictive value, Matthews correlation coefficient, and *F*_1_-score. The Cohen κ was used to assess interrater agreement between the 2 senior radiologists’ initial independent labels for report-level error presence and error categories before consensus adjudication. Intraclass correlation coefficients were used to assess inter- and intraobserver agreement of radiologists’ Likert ratings. All statistical analyses and visualizations were performed using Python (version 3.11.4; Python Software Foundation); Microsoft PowerPoint (version 2407) was used for figure assembly. Statistical significance was defined as *P*<.05.

### Ethical Considerations

This retrospective study was approved by the institutional review boards of center 1 (IPJ2025202) and center 2 (2025kmykdx6f157). Informed consent was waived due to the retrospective design. No protected health information was provided to the LLMs. This study was reported in accordance with the STARD-AI (Standards for Reporting of Diagnostic Accuracy Studies–AI) reporting guidelines.

## Results

### Study Characteristics and Model Screening

The main study dataset comprised 95,582 real-world radiology report texts from the 3 primary imaging modalities: x-ray (n=10,036, 10.5%), CT (n=67,215, 70.3%), and MRI (n=18,331, 19.2%). Among these, 95.4% (91,151/95,582) were error free, and 4.6% (4431/95,582) contained at least one confirmed error (Figure 1). Instance-level analyses included 95,908 labeled instances, comprising 91,192 error-free labels and 4716 true error instances. For 10,000 randomly selected reports (x-ray: 3000; CT: 4000; MRI: 3000), Qwen3 demonstrated superior error detection accuracy (0.96), establishing it as the optimal model for subsequent analysis (Figure S1 in Multimedia Appendix 1).

### Performance of the LLM in Error Detection for Free-Text Radiology Reports

Qwen3 demonstrated modality adaptability and anatomical region robustness in exact label error detection for free-text radiology reports (Figure 2). Across 95,908 labeled instances, the model achieved accuracy of 0.99, balanced accuracy of 0.98, macroprecision of 0.91, macro–*F*_1_-score of 0.94, and weighted *F*_1_-score of 0.99. Performance remained high across radiology reports originating from the 3 imaging modalities, with weighted *F*_1_-score values of 1.00 for x-ray, 1.00 for CT, and 0.99 for MRI. The error rate was 0.5% (52/10,089) for x-ray, 0.6% (368/67,402) for CT, and 1.2% (224/18,417) for MRI, suggesting relatively more false classifications in the longer MRI reports.

**Figure 2.**
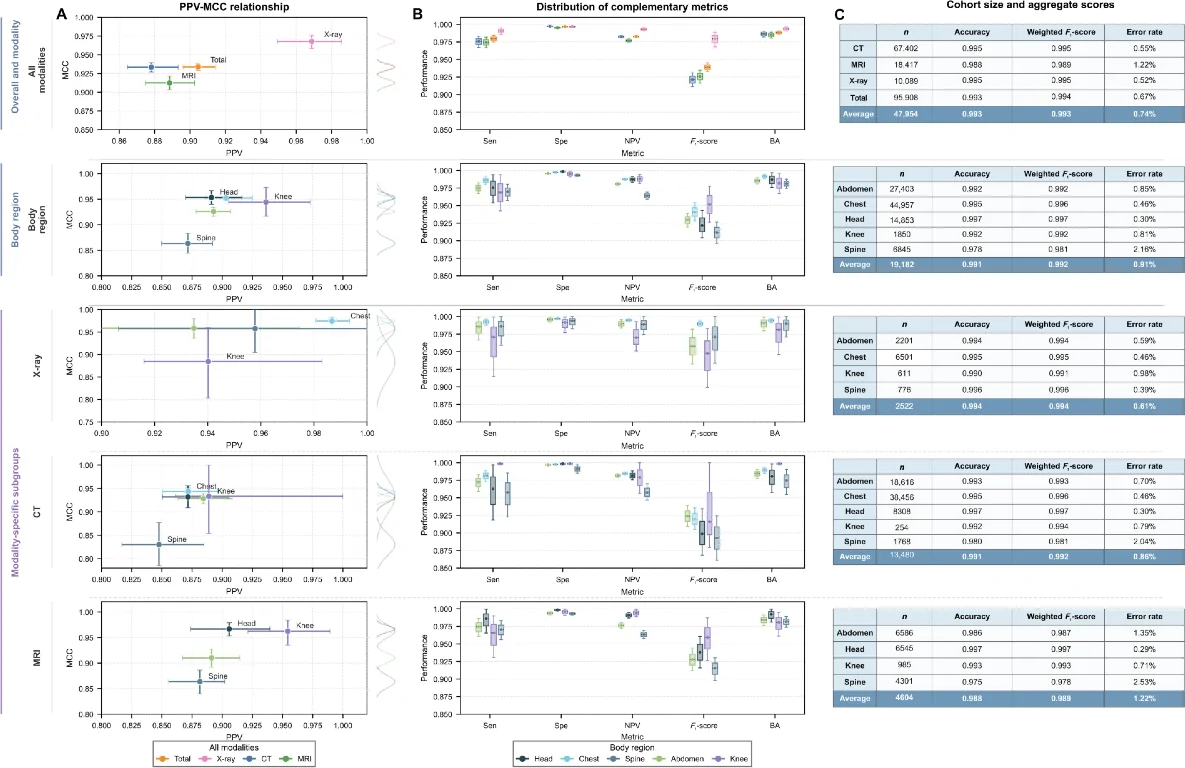
Performance of Qwen3 in radiology report error detection. BA: balanced accuracy; CT: computed tomography; MCC: Matthews correlation coefficient; MRI: magnetic resonance imaging; NPV: negative predictive value; PPV: positive predictive value; Sen: sensitivity; Spe: specificity.

Model discrimination remained stable across anatomical regions (Figure 2). Weighted *F*_1_-score was 1.00 for head, 1.00 for chest, 0.99 for abdomen, 0.98 for spine, and 0.99 for knee. The highest error rate was observed in spine reports (148/6845, 2.2%), whereas head and chest reports had lower error rates of 0.3% (44/14,853) and 0.5% (205/44,957), respectively. These findings indicate that spine reports were more prone to false classifications, consistent with their greater narrative complexity.

In joint stratification by modality and anatomical region, performance remained high but showed clinically relevant heterogeneity (Figure 2). X-ray chest reports showed a weighted *F*_1_-score of 1.00 and an error rate of 0.46%. CT spine and MRI spine reports were more challenging, with weighted *F*_1_-score values of 0.98 and error rates of 2% (36/1768) and 2.5% (109/4301), respectively.

### Performance of the LLM Across Error Types and Granularities

The results of the error type and granularity analysis are shown in Figure 3. Type 2 factual errors were detected more reliably than type 1 interpretive errors. In the primary cohort, type 1 errors achieved accuracy of 0.96, balanced accuracy of 0.95, macroprecision of 0.98, and macro–*F*_1_-score of 0.96. Type 2 errors achieved accuracy of 1.00, balanced accuracy of 1.00, macroprecision of 1.00, and macro–*F*_1_-score of 1.00.

Subtype-level analysis confirmed this pattern. Omission errors (subtype 1) had a precision of 0.83, recall of 0.96, and *F*_1_-score of 0.89; inconsistency errors (subtype 2) had a precision of 0.75, recall of 0.93, and *F*_1_-score of 0.83. Factual subtypes performed better: laterality confusion (subtype 3) had an *F*_1_-score of 0.94; semantic errors (subtype 4) had an *F*_1_-score of 0.98; and other factual errors (subtype 5) had precision, recall, and *F*_1_-score of 1.00. Tables S1 to S4 in Multimedia Appendix 1 provide additional hierarchical and fine-grained classification results.

**Figure 3.**
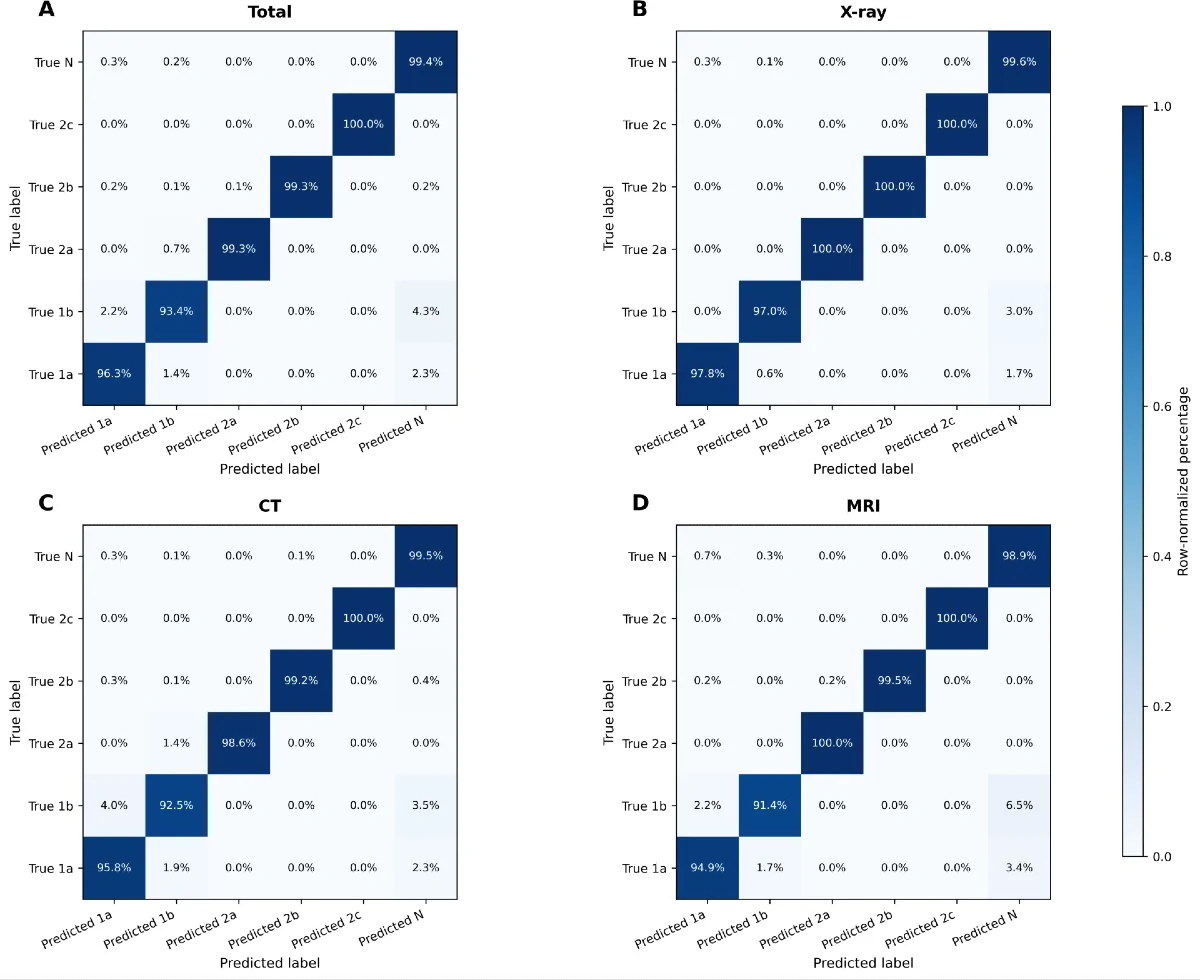
Confusion matrices of predicted versus true error labels. (A) Total cohort; (B) x-ray; (C) CT; and (D) MRI. Values are row-normalized percentages. N indicates no error. 1a: omission errors; 1b: inconsistency errors; 2a: laterality confusion; 2b: semantic errors; 2c: other errors; CT: computed tomography; MRI: magnetic resonance imaging.

### Comparison of Error Identification Between the LLM and Radiologists

An error-enriched, modality-stratified challenge set of 300 radiology reports randomly sampled from expert-confirmed error-positive reports (100 each of x-ray, CT, and MRI examinations) was independently evaluated by 3 radiologists with varying levels of experience and Qwen3. The reports contained 335 expert-confirmed error instances, including 116 x-ray instances, 107 CT instances, and 112 MRI instances (Figures 4A and 4B). The results for report-level error detection sensitivity and report-level exact set match are provided in Tables S5 and S6 in Multimedia Appendix 1. Qwen3 correctly classified 320 of 335 error instances (0.96) compared with 223 of 335 (0.67), 178 of 335 (0.53), and 152 of 335 (0.45) for Drs A, B, and C, respectively. By modality, Qwen3 achieved exact category recall rates of 0.95 for x-ray, 0.96 for CT, and 0.96 for MRI. The corresponding rates were 0.72, 0.64, and 0.62 for Dr A; 0.63, 0.51, and 0.45 for Dr B; and 0.52, 0.50, and 0.35 for Dr C, respectively.

When stratified by broad error category, Qwen3 achieved exact category recall rates of 0.94 for type 1 interpretive errors and 0.97 for type 2 factual errors (Figure 4B). The corresponding rates were 0.62 and 0.70 for Dr A, 0.58 and 0.49 for Dr B, and 0.51 and 0.41 for Dr C, respectively.

**Figure 4.**
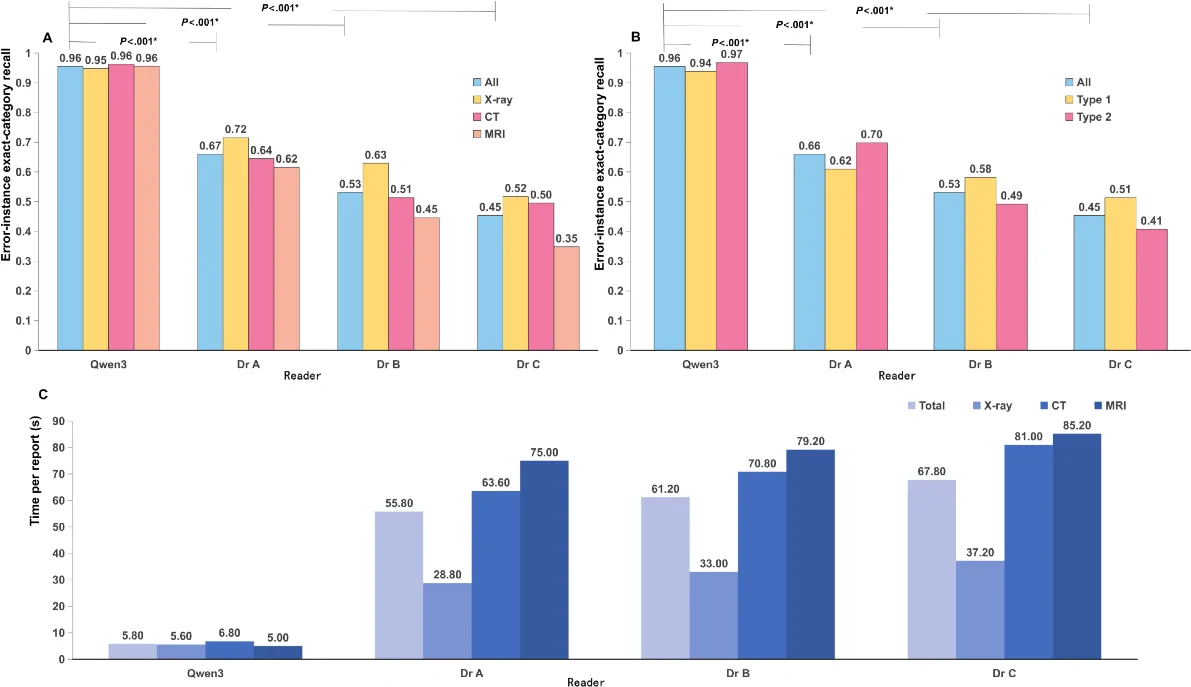
Comparison between Qwen3 and radiologists. (A and B) Error instance exact category recall stratified by imaging modality and error type. Qwen3 demonstrated significantly higher error instance exact category recall than all human reviewers (exact category recall=0.96; *P*<.001), with clinician performance positively correlated with years of experience. (C) Comparison of time consumption between the large language model and radiologists with varying levels of experience revealed that Qwen3 processed each report faster than all human reviewers. CT: computed tomography; MRI: magnetic resonance imaging. *Indicates statistically significant (*P*<.05).

### Time Consumption Comparison

Comparison of review time showed that Qwen3 processed reports faster than all human reviewers (Figure 4C). Mean processing time was 5.80 seconds per report, compared with 55.80 seconds for Dr A, 61.20 seconds for Dr B, and 67.80 seconds for Dr. C. Thus, the model was approximately 9.6 to 11.7 times faster than human reviewers in this constrained text auditing task.

Human review times exhibited a systematic dependence on imaging modality: for x-ray reports, radiologists A, B, and C required 28.80, 33.00, and 37.20 seconds; for CT reports, they required 63.60, 70.80, and 81.00 seconds; and for MRI reports, they required 75.00, 79.20, and 85.20 seconds, respectively. Review time increased markedly with modality complexity, particularly from x-ray to MRI. Human review time was longer for CT and MRI report texts than for x-ray report texts, potentially reflecting differences in report length and textual complexity. In contrast, Qwen3 processed radiology reports efficiently across the 3 imaging modalities, with processing times of 5.60 seconds for x-ray, 6.80 seconds for CT, and 5.00 seconds for MRI, all below 7 seconds per report.

### Analysis of the Error Reasoning Capabilities of the LLM

Figures 5A and 5B show that the LLM’s error reasoning received consistently high evaluations from all 3 radiologists, with an overall mean score of 4.57 (SD 0.86) on the 5-point Likert scale. Stratified by imaging modality, reasoning quality remained high across x-ray (4.63, SD 0.81), CT (4.57, SD 0.84), and MRI (4.52, SD 0.94).

When stratified by error type, a subtle but consistent difference in reasoning quality was observed. The mean score for type 2 (factual) errors was 4.63. This indicates that the model’s explanations for rule-based, discrete errors, such as laterality confusion, unit mislabeling, or typographical slips, were deemed highly reliable and unambiguous by clinicians. In contrast, the mean score for type 1 (interpretive) errors was 4.49, slightly lower but still within the “agree” to “strongly agree” range. This small yet consistent gap suggests that reasoning about interpretive errors such as omissions or description-diagnosis inconsistencies posed a greater challenge for the LLM. Notably, across all modalities, type 2 errors consistently received higher scores than type 1 errors (x-ray: 4.64 vs 4.61; CT: 4.63 vs 4.47; MRI: 4.61 vs 4.40), further confirming that the explicit and well-defined nature of factual errors renders them more amenable to accurate and interpretable algorithmic explanation. Inter- and intraobserver intraclass correlation coefficient values for the Likert ratings exceeded 0.89 (Tables S7 and S8 in Multimedia Appendix 1).

**Figure 5.**
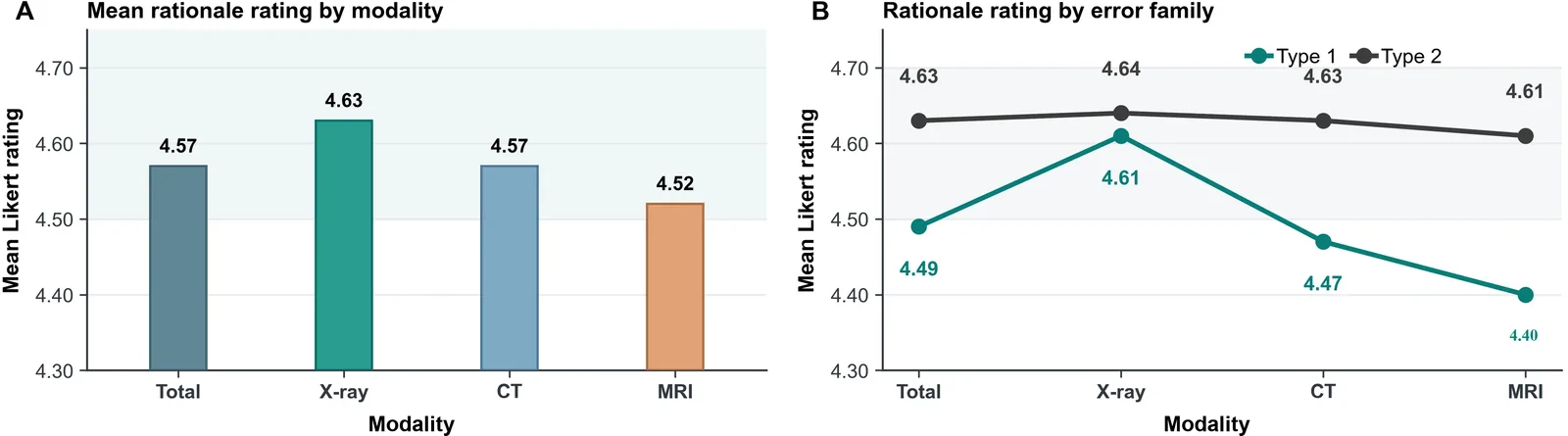
Analysis of error reasoning capabilities in the large language model. (A) Mean Likert rating of the model-generated explanations by imaging modality; (B) mean Likert rating by error family across imaging modalities (Type 1 vs Type 2 errors). CT: computed tomography; MRI: magnetic resonance imaging.

### Cross-Center Validation

The external validation dataset comprised 44,839 real-world radiology reports from center 2, including 5927 (13.2%) x-ray reports, 32,587 (72.7%) CT reports, and 6325 (14.1%) MRI reports. External instance-level analysis included 44,907 labeled instances (Figures 6A-6C). Overall accuracy was 1.00 (95% CI 1.00-1.00), balanced accuracy was 0.94 (95% CI 0.92-0.95), macro-precision was 0.92 (95% CI 0.90-0.94), macro-F1 was 0.93 (95% CI 0.91-0.94), and weighted F1 was 1.00 (95% CI 1.00-1.00). By modality, balanced accuracy was 0.95 (95% CI 0.90-0.98) for x-ray, 0.94 (95% CI 0.91-0.96) for CT, and 0.92 (95% CI 0.88-0.95) for MRI. Error-positive body region analysis showed macro-F1 scores of 0.97 (95% CI 0.94-0.99) for chest, 0.93 (95% CI 0.89-0.97) for head, 0.90 (95% CI 0.86-0.94) for abdomen, and 0.84 (95% CI 0.80-0.89) for spine. Detailed metrics are provided in Tables S9 to S14 and Figure S2 in Multimedia Appendix 1.

**Figure 6.**
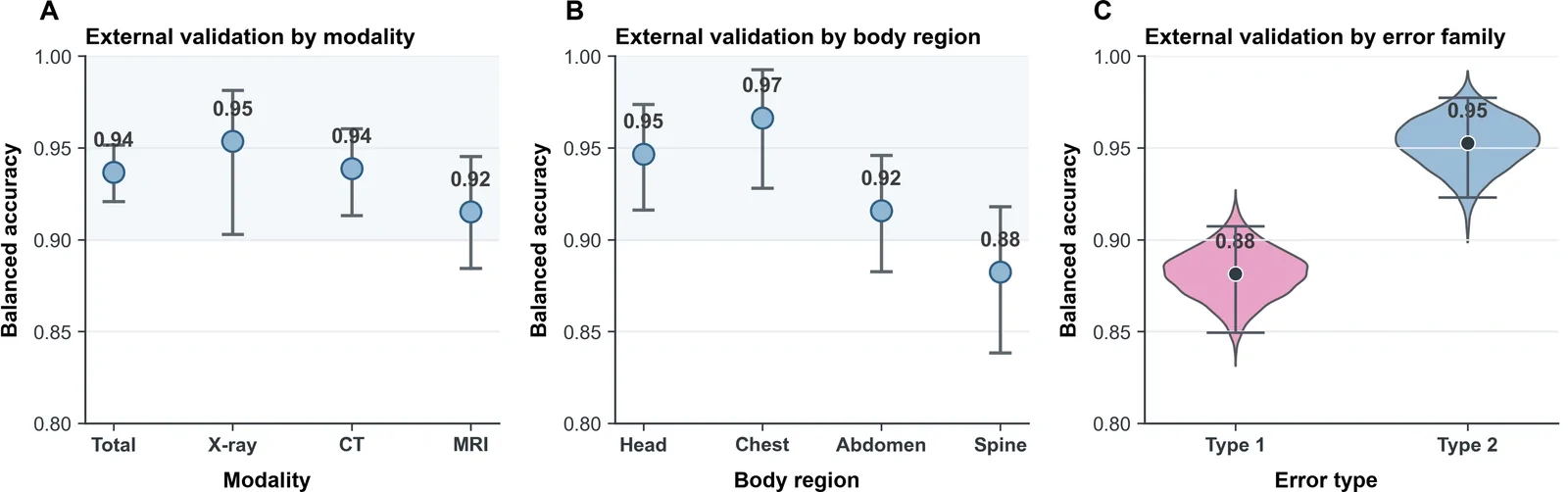
Cross-center validation of Qwen3 in the independent center 2 cohort. (A) Balanced accuracy by imaging modality; (B) balanced accuracy by body region; and (C) balanced accuracy by error type. CT: computed tomography; MRI: magnetic resonance imaging.

### Interrater Agreement on Reference Standard Annotations

Before consensus adjudication, the same 2 senior radiologists showed substantial interrater agreement for report-level error presence at both centers (center 1: Cohen κ=0.783, 95% CI 0.773‐0.792; center 2: Cohen κ=0.850, 95% CI 0.837‐0.863; *P*<.001 in both cases). Overall, positive, and negative agreement were 97.93%, 79.35%, and 98.91% at center 1 and 98.90%, 85.60%, and 99.43%, respectively, at center 2. Among consensus-confirmed error-positive reports, category-specific Cohen κ values ranged from 0.816 to 0.975 at center 1 and from 0.841 to 0.985 at center 2 (*P*<.001; Tables S15 and S16 in Multimedia Appendix 1).

## Discussion

### Principal Findings

This study provides real-world evidence to validate the feasibility and performance of a general-purpose LLM for radiology report error detection in real clinical settings across radiology reports from 3 imaging modalities (x-ray, CT, and MRI), multiple anatomical regions, and 2 institutions. Unlike prior work relying on synthetic datasets [1], our study used a fine-grained, expert-anchored error taxonomy to ensure high ecological validity. Key findings are as follows: (1) Qwen3 showed robust generalizability across radiology reports from the 3 imaging modalities, multiple anatomical regions, and 2 institutions; and (2) in blinded head-to-head comparison, the model achieved higher exact category recall rates than senior radiologists and processed reports nearly 10 times faster while providing clinically credible explanations. These results indicate that general-purpose LLMs can serve as transparent and efficient auditing tools, offering a scalable solution for radiology quality assurance.

### Performance Across Reporting Contexts

The study shows that Qwen3 performs robustly across free-text radiology reports from all 3 imaging modalities, indicating its ability to adapt to diverse reporting structures and error contexts [24]. However, model performance correlated with the linguistic standardization of reports, not with the inherent complexity of the imaging modality [25]. We observed the best performance in highly structured chest x-ray reports. In contrast, spinal CT and MRI reports posed greater challenges due to complex descriptions of degenerative changes and extensive synonym variation (eg, “osteophyte,” “sclerosis,” and “spur”). This performance gradient suggests that scaling model size alone may not resolve domain-specific limitations. Future deployment should incorporate anatomy-specific fine-tuning or context-enhanced prompting strategies [26,27].

### Text Internal Error Types and Model Reasoning

Regarding error taxonomy, Qwen3 demonstrated excellent reliability in detecting rule-based “factual errors” (type 2 errors). These include errors such as laterality confusion and unit mistakes. However, the model showed relatively weaker performance for “interpretive errors” (type 1 errors). These errors require cross-sentence reasoning. This finding suggests that LLMs should help preserve expert cognitive bandwidth for intricate diagnostic tasks [27,28]. Another possible contributor to the lower performance for interpretive errors is that the model operated exclusively on report text and had no access to the corresponding images. Therefore, it could assess the internal consistency and completeness of the report text but could not independently determine whether an imaging finding had been overlooked or misinterpreted. The mismatch exists between the need for deep clinical reasoning and the capabilities of text-only LLMs. It does not reflect a deficiency in medical language understanding itself [29,30].

### Human-AI Comparison and Clinical Implications

Comparisons with radiologists of varying seniority levels revealed the advantages of the LLM. The model maintained high error instance exact category recall while operating significantly faster than human readers. Furthermore, the participating radiologists rated the model-generated explanations favorably, supporting their perceived clarity and clinical plausibility within the evaluated text auditing task. This establishes a foundation of trust for using the model as an assistive quality control tool [31]. These findings support a risk-stratified implementation strategy for clinical practice. High-confidence detections of type 2 errors can trigger automatic alerts for immediate correction. For type 1 errors, we suggest using model outputs as prompts for radiologist review. Furthermore, the model offers advantages in processing speed (less than 7 seconds per report) and cross-center generalization. The model’s short processing time suggests that it may be compatible with high-throughput clinical workflows while adding minimal time burden [20,32]. Its consistent performance across centers further supports its potential applicability in different clinical settings.

Under the workload and token pricing assumptions used in this study, the estimated recurring API cost was approximately US $0.00035 to US $0.00347 per report. Relative to the operational scale of a large tertiary hospital, this represents a modest per-report expenditure. Together with the observed performance of the model in radiology report text quality control, these estimates suggest potential economic feasibility and clinical translational value for the proposed workflow. However, formal cost-effectiveness remains to be established through real-world implementation studies that account for infrastructure, personnel, maintenance, workflow integration, and long-term operational costs.

### Limitations

This study has several limitations. First, the use of Chinese-only reports may limit generalizability to other languages. Second, some interpretive error labels remain subjective despite expert adjudication. Third, this study evaluated only errors identifiable from report text and could not assess image-report concordance or detect errors requiring direct interpretation of the source images. Future studies should evaluate vision language models that jointly incorporate medical images, report text, and relevant clinical context. Fourth, in the human-AI comparison, radiologists did not have access to the source images or patient clinical information, unlike in routine practice. Therefore, LLM performance in clinical image interpretation and routine report review requires further validation. Recorded human review times reflected only the experimental task and not routine clinical workflow. Fifth, model processing times for reports from different imaging modalities may have been affected by input and output text length, API and network latency, and server load. The observed differences should therefore not be interpreted as inherent modality-specific differences in report text processing efficiency under real-world clinical conditions. Finally, we did not categorize error severity based on clinical impact, which warrants further investigation with subspecialty involvement.

### Conclusions

In conclusion, our findings suggest that LLMs can serve as efficient adjunctive tools for detecting prespecified text internal errors in radiology reports, particularly explicit factual errors. Although challenges remain in context-dependent interpretive reasoning, the model’s efficient report text processing, scalability, and decision logic that were judged acceptable by radiologists suggest that it may support text-level quality assurance amid increasing clinical workloads.

## Supplementary material

10.2196/106250 Multimedia Appendix 1 Quality-check prompt, examples of model error detection, and supplementary figures and tables (Figures S1 and S2; Tables S1-S16).

## Abbreviations

CT: computed tomography
LLM: large language model
MRI: magnetic resonance imaging
STARD-AI: Standards for Reporting of Diagnostic Accuracy Studies–Artificial Intelligence
