# Supplementary material for: Large Language Model–Assisted Radiology Report Quality Assurance: Real-World Multicenter Evaluation
Source: J Med Internet Res. 2026 Oct 28;28:e106250. doi: 10.2196/106250 (PMC13717678; doi:10.2196/106250)
Supplement: Supplementary file 1 [file jmir-v28-e106250-s001.docx]

**Supplemental Appendix S1**

**Radiology Report Quality Check Prompt**

\## Role Definition

You are a radiology report quality control expert.

You perform structured text checks—no medical interpretation, just review of report compliance.

\## Task Instructions

Review input exam reports using these rules:

\## Check Rules

1. Basic Guidelines
A. Only output pre-defined format conclusions. No extra explanations.
B. If a check item has no issues or is not applicable, skip it.
C. No medical interpretation or diagnostic opinions.
D. Lesion size can be: 11.0cm×7.5cm×11.3cm / 11.0×7.5×11.3cm / 11.0×7.5cm. These show size—not multiplication. All formats are acceptable (mix freely).
E. Double-check: missing items, inconsistencies (within/between sections), positional errors. Ensure no omissions or mistakes.
2. Content Check Dimensions and Error Categories

Type 1 Interpretive errors:

(1a) Omission: Check if corresponding imaging descriptions or diagnoses are missing—based on "Exam Items," "Imaging Findings," and "Diagnostic Impressions."

(1b) Inconsistency: Imaging descriptions conflict with diagnoses. E.g., imaging notes "clear fracture line," but diagnosis says "no definite fracture line."

Type 2 Factual Errors:

(2a) Laterality Confusion: Wrong side/orientation (e.g., recording right as left, or lateral as medial).

(2b) Semantic Errors: Unintentional wrong words/phrases—improper terminology, incorrect substitutions, inserted content, or typos.

(2c) Other Errors: Errors not in the above categories—wrong diagnosis numbers, unit mistakes (e.g., cm vs. mm), punctuation/format issues.

Reports with no errors: Classified as (N).

3. Output Specifications

If no errors are detected, report as: [(N) No issues]

If errors are detected, report as:

[Report Questionable]

[Error dimension, specific error type]: Detailed error description;

Reasoning process: Require checking each verification dimension to ensure no omissions or errors.

Reference Example:

[Exam Item] Upper and middle abdominal spiral CT plain scan

[Imaging Findings] Liver morphology/size normal; no abnormal density foci. Gallbladder not enlarged; no wall thickening. No abnormal foci in gallbladder. Pancreas: no abnormal foci. Spleen not enlarged; no abnormal foci. Bilateral adrenal glands: no nodules/masses. Bilateral kidneys: symmetric; no abnormal foci.

[Diagnostic Impression] No obvious abnormalities on plain scan of liver, gallbladder, pancreas, spleen, bilateral adrenal glands, or bilateral kidneys.

Qwen 3 Output:

[(N) No issues]

Analysis Process:

Type 1: Interpretive Errors

(1a) Omission:

Exam: non-contrast CT of upper/mid abdomen.

Findings cover all key organs: liver, gallbladder, pancreas, spleen, bilateral adrenal glands, and kidneys.

All structures are described as “no abnormality.”

Impression states “no significant abnormality,” matching the findings exactly.

No positive findings were reported, so no additional diagnoses are needed.

→ No omission.

(1b) Inconsistency:

All findings use consistent negative phrasing (e.g., “no abnormal density,” “no wall thickening,” “no nodules or masses”).

Impression conclusion aligns: “no significant abnormality.”

→ No inconsistency.

Type 2: Factual Errors

(2a) Laterality error:

Findings refer to “bilateral kidneys” and “bilateral adrenal glands.”

Impression uses the same terms.

No unilateral or side-specific labels are present.

→ Not applicable; no error.

(2b) Semantic error:

Phrases like “no abnormal density,” “no thickening,” and “no nodules or masses” are standard.

“No significant abnormality” uses “significant” appropriately—as a clinically accepted qualifier.

No misspellings, word substitutions, or unintended insertions.

→ No semantic error.

(2c) Other errors:

Only one impression statement is given, so numbering is not required.

No measurements → no unit errors.

Punctuation is correct and minimal (no terminal period is acceptable in concise reporting).

→ No other errors.

Conclusion:

The report is complete, well-structured, and internally consistent. Findings and impression align strictly. It meets radiology reporting quality standards.

Final verdict: [(N) No issues].

**Supplemental Appendix S2**

**Report Error Detection Examples:**

**Case 1:**

[Exam Item] Upper and middle abdominal spiral CT plain scan

[Imaging Findings] Liver morphology/size normal; no abnormal density foci. Gallbladder not enlarged; no wall thickening. Pancreas: no abnormal density foci. Spleen not enlarged; no abnormal density foci. Bilateral adrenal glands: no nodules/masses. Bilateral kidneys symmetric; right kidney has a ~0.5×0.5cm roundish low-density focus

[Diagnostic Impression] 1. Possible small renal cyst in the right kidney.

Qwen 3 Output:

[(N) No issues]

Analysis Process:

(1a) Omission:

Lesion location, number, size, and enhancement features are all reported.

(1b) Inconsistency:

“Low-density lesion” is compatible with a cyst.

No direct contradiction between findings and impression.

(2a) Laterality confusion:

Both findings and impression correctly specify “right kidney.”

(2b) Semantic error:

The term “possible cyst” appropriately reflects diagnostic uncertainty in a non-contrast scan.

Wording is clinically appropriate.

(2c) Other errors:

Impression numbering, punctuation, and units are all correct.

Conclusion:

All required elements are present and consistent.

Final classification: [(N) No issues].

**Case 2**

[Exam Item] Chest spiral CT plain scan

[Imaging Findings] Bilateral pulmonary markings: normal course and distribution.

Multiple solid nodules in the superior lingular segment of the left upper lobe and dorsal segment of the left lower lobe. The largest nodule is in the left upper lobe’s superior lingular segment (IM257), measuring ~3×3mm. No exudates, nodules, or masses in the rest of the lungs. No pleural effusion bilaterally. Heart not enlarged; hilar structures clear. No definite enlarged lymph nodes in the mediastinum.

[Diagnostic Impression]

Left upper lobe superior lingular segment solid micro-nodule. Lung-RADS 2, likely benign.

Qwen 3 Output:

[Report flagged]

[Type 1, (1a) omission] The diagnostic impression omits the multiple solid nodules in the left lower lobe (posterior segment), as described in the imaging findings.

Analysis Process:

I carefully reviewed the structured chest CT report to check compliance with reporting standards. Each error category was verified twice to avoid oversight.

Omission check:

Exam: chest CT (non-contrast, helical).

Imaging findings: multiple solid nodules in both the left upper lobe (lingular segment) and left lower lobe (posterior segment).

Diagnostic impression: only mentions nodules in the “left upper lung” and assigns Lung-RADS 2.

→ The left lower lobe nodules are not included. This is a clear omission.

(1b) Inconsistency:

Both findings and impression agree that nodules exist. No contradiction.

(2a) Laterality confusion:

“Left lung” is consistently used. No right–left mix-up.

(2b) Semantic error:

“Lung-RADS” is spelled correctly and used appropriately.

(2c) Other errors:

Size “3mm×3mm” follows accepted format.

Numbering and punctuation are correct.

Conclusion:

The diagnostic impression fails to reflect all positive findings—specifically, the nodules in the left lower lobe posterior segment.

This constitutes a Type 1, (1a) omission error.

**Supplemental Figure S1 Comparison of error detection performance among five large language models across 10,000 radiology reports.**

**Supplementary Table S1. Exact-label multiclass performance with 95% confidence intervals by modality and body part**

| **Group** | **Total instances** | **Accuracy** | **Balanced accuracy** | **Macro-precision** | **Macro-F1** | **Weighted-F1** |
| --- | --- | --- | --- | --- | --- | --- |
| Overall | 95908 | 0.993 (0.993-0.994) | 0.980 (0.975-0.984) | 0.905 (0.897-0.913) | 0.939 (0.934-0.944) | 0.994 (0.993-0.994) |
| X-ray | 10089 | 0.995 (0.993-0.996) | 0.991 (0.985-0.995) | 0.969 (0.948-0.985) | 0.979 (0.967-0.988) | 0.995 (0.993-0.996) |
| CT | 67402 | 0.995 (0.994-0.995) | 0.976 (0.968-0.983) | 0.878 (0.862-0.895) | 0.921 (0.910-0.932) | 0.995 (0.994-0.995) |
| MRI | 18417 | 0.988 (0.986-0.990) | 0.975 (0.967-0.983) | 0.888 (0.875-0.903) | 0.926 (0.916-0.936) | 0.989 (0.987-0.990) |
| Head | 14853 | 0.997 (0.996-0.998) | 0.976 (0.951-0.995) | 0.891 (0.867-0.914) | 0.922 (0.900-0.941) | 0.997 (0.997-0.998) |
| Chest | 44957 | 0.995 (0.995-0.996) | 0.986 (0.981-0.991) | 0.903 (0.886-0.926) | 0.941 (0.928-0.955) | 0.996 (0.995-0.996) |
| Abdomen | 27403 | 0.992 (0.991-0.993) | 0.975 (0.967-0.982) | 0.893 (0.880-0.909) | 0.930 (0.921-0.940) | 0.992 (0.991-0.993) |
| Spine | 6845 | 0.978 (0.975-0.982) | 0.970 (0.957-0.981) | 0.872 (0.848-0.892) | 0.912 (0.896-0.926) | 0.981 (0.978-0.983) |
| Knee | 1850 | 0.992 (0.988-0.996) | 0.969 (0.937-0.991) | 0.936 (0.908-0.969) | 0.952 (0.926-0.975) | 0.992 (0.988-0.996) |

**Supplementary Table S2. Exact-label performance with 95% confidence intervals in non-zero modality-by-body-part subgroups**

| **Group** | **Total instances** | **Balanced accuracy** | **Macro-precision** | **Macro-F1** | **Weighted-F1** |
| --- | --- | --- | --- | --- | --- |
| X-ray-Chest | 6501 | 0.992 (0.987-0.997) | 0.987 (0.980-0.992) | 0.990 (0.985-0.994) | 0.995 (0.994-0.997) |
| X-ray-Abdomen | 2201 | 0.986 (0.965-0.999) | 0.935 (0.900-0.972) | 0.958 (0.934-0.980) | 0.994 (0.991-0.997) |
| X-ray-Spine | 776 | 0.986 (0.959-1.000) | 0.958 (0.910-1.000) | 0.971 (0.938-1.000) | 0.996 (0.991-1.000) |
| X-ray-Knee | 611 | 0.971 (0.915-0.999) | 0.940 (0.912-0.983) | 0.948 (0.907-0.983) | 0.991 (0.984-0.997) |
| CT-Head | 8308 | 0.963 (0.911-0.996) | 0.872 (0.850-0.902) | 0.899 (0.863-0.931) | 0.997 (0.997-0.998) |
| CT-Chest | 38456 | 0.981 (0.973-0.989) | 0.872 (0.851-0.898) | 0.920 (0.905-0.935) | 0.996 (0.995-0.996) |
| CT-Abdomen | 18616 | 0.973 (0.960-0.984) | 0.884 (0.862-0.904) | 0.924 (0.908-0.937) | 0.993 (0.992-0.994) |
| CT-Spine | 1768 | 0.958 (0.927-0.984) | 0.848 (0.813-0.887) | 0.893 (0.862-0.924) | 0.981 (0.976-0.987) |
| CT-Knee | 254 | 0.999 (0.997-1.000) | 0.889 (0.861-1.000) | 0.916 (0.879-1.000) | 0.994 (0.987-1.000) |
| MRI-Head | 6545 | 0.986 (0.968-0.999) | 0.906 (0.877-0.943) | 0.938 (0.917-0.961) | 0.997 (0.996-0.998) |
| MRI-Abdomen | 6586 | 0.975 (0.963-0.986) | 0.891 (0.868-0.914) | 0.928 (0.914-0.943) | 0.987 (0.985-0.990) |
| MRI-Spine | 4301 | 0.970 (0.955-0.983) | 0.882 (0.859-0.904) | 0.916 (0.899-0.932) | 0.978 (0.974-0.982) |
| MRI-Knee | 985 | 0.966 (0.928-0.994) | 0.954 (0.921-0.989) | 0.959 (0.927-0.989) | 0.993 (0.988-0.997) |

**Supplementary Table S3. Performance across Type 1 and Type 2 error types**

| **Group** | **Total instances** | **Accuracy** | **Balanced accuracy** | **Macro-precision** | **Macro-F1** |
| --- | --- | --- | --- | --- | --- |
| Type 1 | 2260 | 0.955 (0.947-0.962) | 0.948 (0.936-0.958) | 0.975 (0.967-0.983) | 0.961 (0.953-0.969) |
| Type 2 | 2456 | 0.996 (0.994-0.998) | 0.996 (0.990-0.999) | 0.998 (0.993-1.000) | 0.997 (0.993-0.999) |

| **Subtype** | **Support** | **Predicted** | **Precision** | **Recall** | **F1** |
| --- | --- | --- | --- | --- | --- |
| 1a | 1682 | 1951 | 0.830 (0.815-0.845) | 0.963 (0.953-0.971) | 0.891 (0.881-0.901) |
| 1b | 578 | 725 | 0.745 (0.718-0.773) | 0.934 (0.915-0.955) | 0.829 (0.810-0.849) |
| 2a | 149 | 167 | 0.886 (0.846-0.931) | 0.993 (0.980-1.000) | 0.937 (0.913-0.961) |
| 2b | 1221 | 1254 | 0.967 (0.957-0.977) | 0.993 (0.989-0.998) | 0.980 (0.974-0.986) |
| 2c | 1086 | 1086 | 1.000 (1.000-1.000) | 1.000 (1.000-1.000) | 1.000 (1.000-1.000) |
| No error | 91192 | 90725 | 0.999 (0.999-0.999) | 0.994 (0.994-0.995) | 0.997 (0.996-0.997) |

**Supplementary Table S4. Overall exact-label subtype performance with 95% confidence intervals**

**Supplementary Table S5. Report-level error-detection sensitivity.**

| **Modality** | **Qwen 3** | **Doctor A** | **Doctor B** | **Doctor C** |
| --- | --- | --- | --- | --- |
| All modalities | 294/300 (0.98) | 256/300 (0.85) | 220/300 (0.73) | 218/300 (0.73) |
| X-ray | 100/100 (1.00) | 88/100 (0.88) | 82/100 (0.82) | 73/100 (0.73) |
| CT | 96/100 (0.96) | 87/100 (0.87) | 69/100 (0.69) | 99/100 (0.99) |
| MRI | 98/100 (0.98) | 81/100 (0.81) | 69/100 (0.69) | 46/100 (0.46) |

*Values are n/N. A report was counted as detected when at least one prespecified error category was identified.*

**Supplementary Table S6. Report-level exact-set match.**

| **Modality** | **Qwen 3** | **Doctor A** | **Doctor B** | **Doctor C** |
| --- | --- | --- | --- | --- |
| All modalities | 286/300 (0.95) | 197/300 (0.66) | 151/300 (0.50) | 134/300 (0.45) |
| X-ray | 94/100 (0.94) | 70/100 (0.70) | 60/100 (0.60) | 50/100 (0.50) |
| CT | 96/100 (0.96) | 65/100 (0.65) | 50/100 (0.50) | 50/100 (0.50) |
| MRI | 96/100 (0.96) | 62/100 (0.62) | 41/100 (0.41) | 34/100 (0.34) |

*Values are n/N. Exact-set match required the complete predicted error-category set to equal the expert reference set.*

**Supplementary Table S7. Inter-rater reliability of radiologists’ Likert ratings of LLM-generated explanations.**

| **Dataset** | **n** | **ICC (95% CI)** | ***P* value*** |
| --- | --- | --- | --- |
| All reports | 300 | 0.906 (0.860–0.936) | <0.001***** |
| X-ray | 100 | 0.910 (0.789–0.958) | <0.001***** |
| CT | 100 | 0.892 (0.794–0.938) | <0.001***** |
| MRI | 100 | 0.914 (0.827–0.959) | <0.001***** |

CI, confidence interval; ICC, intraclass correlation coefficient; LLM, large language model. * Statistically significant (*P* <0.05).

**Supplementary Table S8. Intra-rater reliability of the repeat ratings for each radiologist, overall and by imaging modality.**

| **Radiologist** | **Dataset** | **n** | **ICC (95% CI)** | ***P* value** |
| --- | --- | --- | --- | --- |
| **Doctor A** | Total | 90 | 0.943 (0.869-0.979) | <0.001***** |
|  | X-ray | 30 | 0.948 (0.535-1.000) | <0.001***** |
|  | CT | 30 | 0.972 (0.897-1.000) | <0.001***** |
|  | MRI | 30 | 0.896 (0.256-0.979) | <0.001***** |
| **Doctor B** | Total | 90 | 0.948 (0.898-0.978) | <0.001***** |
|  | X-ray | 30 | 0.958 (0.853-1.000) | <0.001***** |
|  | CT | 30 | 0.924 (0.749-0.985) | <0.001***** |
|  | MRI | 30 | 0.972 (0.958-1.000) | <0.001***** |
| **Doctor C** | Total | 90 | 0.958 (0.913-0.984) | <0.001***** |
|  | X-ray | 30 | 0.972 (0.920-1.000) | <0.001***** |
|  | CT | 30 | 0.967 (0.794-1.000) | <0.001***** |
|  | MRI | 30 | 0.932 (0.777-0.987) | <0.001***** |

CI, confidence interval; ICC, intraclass correlation coefficient. * Statistically significant (*P* <0.05).

**Supplementary Table S9. External validation cohort distribution of true error-positive reports**

| **Group** | **True error-positive reports** | **Share of all error-positive reports** |
| --- | --- | --- |
| Overall | 1020 | 100.0% |
| X-ray | 301 | 29.5% |
| CT | 402 | 39.4% |
| MRI | 317 | 31.1% |

| **Group** | **Subtype** | **Count** | **Within-group share** |
| --- | --- | --- | --- |
| Overall | 1a | 248 | 22.8% |
|  | 1b | 294 | 27.0% |
|  | 2a | 35 | 3.2% |
|  | 2b | 432 | 39.7% |
|  | 2c | 78 | 7.2% |
| X-ray | 1a | 82 | 25.7% |
|  | 1b | 89 | 27.9% |
|  | 2a | 9 | 2.8% |
|  | 2b | 122 | 38.2% |
|  | 2c | 17 | 5.3% |
| CT | 1a | 121 | 28.1% |
|  | 1b | 114 | 26.5% |
|  | 2a | 18 | 4.2% |
|  | 2b | 150 | 34.9% |
|  | 2c | 27 | 6.3% |
| MRI | 1a | 45 | 13.3% |
|  | 1b | 91 | 26.9% |
|  | 2a | 8 | 2.4% |
|  | 2b | 160 | 47.3% |
|  | 2c | 34 | 10.1% |

**Supplementary Table S10. Subtype distribution of true error types and granularities**

**Supplementary Table S11. Exact-label multiclass performance with 95% confidence intervals by modality**

| **Group** | **Total instances** | **ACC** | **Balanced accuracy** | **Macro-Precision** | **Macro-F1** | **Weighted-F1** |
| --- | --- | --- | --- | --- | --- | --- |
| Overall | 44907 | 0.998 (0.998-0.998) | 0.937 (0.921-0.952) | 0.920 (0.896-0.941) | 0.927 (0.908-0.943) | 0.998 (0.998-0.998) |
| X-ray | 5946 | 0.997 (0.996-0.998) | 0.954 (0.903-0.981) | 0.929 (0.877-0.974) | 0.940 (0.892-0.972) | 0.997 (0.996-0.998) |
| CT | 32615 | 0.999 (0.998-0.999) | 0.939 (0.913-0.960) | 0.919 (0.884-0.955) | 0.927 (0.895-0.955) | 0.999 (0.998-0.999) |
| MRI | 6346 | 0.995 (0.993-0.996) | 0.915 (0.884-0.945) | 0.913 (0.883-0.939) | 0.913 (0.882-0.939) | 0.995 (0.993-0.997) |

**Supplementary Table S12. Exact-label multiclass performance with 95% confidence intervals by body part**

| **Group** | **Error-positive instances** | **ACC** | **Balanced accuracy** | **Macro-Precision** | **Macro-F1** | **Weighted-F1** |
| --- | --- | --- | --- | --- | --- | --- |
| Head | 222 | 0.937 (0.905-0.966) | 0.947 (0.916-0.974) | 0.916 (0.866-0.974) | 0.928 (0.885-0.970) | 0.940 (0.910-0.969) |
| Chest | 271 | 0.978 (0.956-0.993) | 0.966 (0.928-0.993) | 0.980 (0.961-0.995) | 0.973 (0.944-0.992) | 0.978 (0.956-0.993) |
| Abdomen | 276 | 0.913 (0.882-0.942) | 0.916 (0.883-0.946) | 0.895 (0.851-0.943) | 0.904 (0.861-0.940) | 0.913 (0.883-0.943) |
| Spine | 318 | 0.865 (0.827-0.903) | 0.882 (0.838-0.918) | 0.820 (0.769-0.887) | 0.843 (0.795-0.893) | 0.865 (0.828-0.901) |

**Supplementary Table S13. Performance across Type 1 and Type 2 error types and granularities discrimination**

| **Class** | **Support** | **Predicted** | **Precision** | **Recall** | **F1** |
| --- | --- | --- | --- | --- | --- |
| Type 1 | 542 | 545 | 0.983 (0.973-0.993) | 0.989 (0.980-0.996) | 0.986 (0.979-0.993) |
| Type 2 | 545 | 542 | 0.987 (0.977-0.996) | 0.982 (0.969-0.992) | 0.984 (0.976-0.992) |
| 1a | 248 | 247 | 0.879 (0.840-0.916) | 0.875 (0.831-0.911) | 0.877 (0.843-0.905) |
| 1b | 294 | 289 | 0.903 (0.870-0.934) | 0.888 (0.847-0.924) | 0.895 (0.866-0.919) |
| 2a | 35 | 39 | 0.872 (0.778-0.971) | 0.971 (0.914-1.000) | 0.919 (0.857-0.972) |
| 2b | 432 | 421 | 0.988 (0.977-0.998) | 0.963 (0.944-0.979) | 0.975 (0.965-0.985) |
| 2c | 78 | 75 | 0.960 (0.920-1.000) | 0.923 (0.859-0.974) | 0.941 (0.901-0.974) |

**Supplementary Figure S2. Six-class confusion matrices by modality.**

**Supplementary Table S14. Modality-specific exact-label subtype performance with 95% confidence intervals**

| **Group** | **Subtype** | **Support** | **Predicted** | **Precision** | **Recall** | **F1** |
| --- | --- | --- | --- | --- | --- | --- |
| X-ray | 1a | 82 | 78 | 0.949 (0.893-0.989) | 0.902 (0.833-0.962) | 0.925 (0.881-0.962) |
|  | 1b | 89 | 94 | 0.904 (0.842-0.962) | 0.955 (0.908-0.989) | 0.929 (0.887-0.965) |
|  | 2a | 9 | 11 | 0.727 (0.436-1.000) | 0.889 (0.585-1.000) | 0.800 (0.533-0.964) |
|  | 2b | 122 | 120 | 0.992 (0.970-1.000) | 0.975 (0.949-1.000) | 0.983 (0.966-0.996) |
|  | 2c | 17 | 17 | 1.000 (1.000-1.000) | 1.000 (1.000-1.000) | 1.000 (1.000-1.000) |
| CT | 1a | 121 | 122 | 0.877 (0.818-0.930) | 0.884 (0.826-0.941) | 0.881 (0.836-0.922) |
|  | 1b | 114 | 111 | 0.892 (0.833-0.949) | 0.868 (0.812-0.932) | 0.880 (0.835-0.927) |
|  | 2a | 18 | 22 | 0.818 (0.641-0.958) | 1.000 (1.000-1.000) | 0.900 (0.781-0.979) |
|  | 2b | 150 | 148 | 0.966 (0.935-0.993) | 0.953 (0.917-0.986) | 0.960 (0.936-0.982) |
|  | 2c | 27 | 26 | 0.962 (0.868-1.000) | 0.926 (0.807-1.000) | 0.943 (0.857-1.000) |
| MRI | 1a | 45 | 52 | 0.692 (0.560-0.815) | 0.800 (0.667-0.906) | 0.742 (0.630-0.832) |
|  | 1b | 91 | 88 | 0.875 (0.799-0.941) | 0.846 (0.765-0.922) | 0.860 (0.794-0.912) |
|  | 2a | 8 | 8 | 1.000 (1.000-1.000) | 1.000 (1.000-1.000) | 1.000 (1.000-1.000) |
|  | 2b | 160 | 158 | 0.975 (0.949-0.994) | 0.963 (0.934-0.988) | 0.969 (0.949-0.988) |
|  | 2c | 34 | 32 | 0.938 (0.846-1.000) | 0.882 (0.767-0.974) | 0.909 (0.826-0.971) |

**Supplementary Table S15. Inter-rater Agreement for Report-Level Error Presence at Centers 1 and 2**

| **Center** | **Overall agreement, %** | **Positive agreement, %** | **Negative agreement, %** | **Kappa (95% CI)** | ***P* value** |
| --- | --- | --- | --- | --- | --- |
| **Center 1** | 97.93 | 79.35 | 98.91 | 0.783 (0.773-0.792) | <0.001^*^ |
| **Center 2** | 98.90 | 85.60 | 99.43 | 0.850 (0.837-0.863) | <0.001^*^ |

CI, confidence interval; * Statistically significant (*P* <0.05).

**Supplementary Table S16. Category-Specific Inter-rater Agreement Among Consensus-Confirmed Error-Positive Reports at Centers 1 and 2**

| **Center** | **Error category** | **Agreement, %** | **Kappa (95% CI)** | ***P* value** |
| --- | --- | --- | --- | --- |
| **Center 1** | Type 1 interpretive error | 90.97 | 0.816 (0.799-0.833) | <0.001^*^ |
|  | Type 2 factual error | 96.39 | 0.928 (0.917-0.939) | <0.001^*^ |
|  | 1a Omission | 92.82 | 0.838 (0.820-0.855) | <0.001^*^ |
|  | 1b Inconsistency | 97.49 | 0.876 (0.853-0.899) | <0.001^*^ |
|  | 2a Laterality confusion | 99.84 | 0.975 (0.957-0.994) | <0.001^*^ |
|  | 2b Semantic error | 97.90 | 0.946 (0.935-0.957) | <0.001^*^ |
|  | 2c Other error | 98.28 | 0.953 (0.942-0.963) | <0.001^*^ |
| **Center 2** | Type 1 interpretive error | 92.16 | 0.841 (0.807-0.874) | <0.001^*^ |
|  | Type 2 factual error | 97.84 | 0.957 (0.939-0.975) | <0.001^*^ |
|  | 1a Omission | 95.59 | 0.869 (0.831-0.906) | <0.001^*^ |
|  | 1b Inconsistency | 95.98 | 0.889 (0.856-0.922) | <0.001^*^ |
|  | 2a Laterality confusion | 99.90 | 0.985 (0.956-1.000) | <0.001^*^ |
|  | 2b Semantic error | 98.14 | 0.962 (0.944-0.979) | <0.001^*^ |
|  | 2c Other error | 99.71 | 0.978 (0.953-1.000) | <0.001^*^ |

CI, confidence interval; * Statistically significant (*P* <0.05).
